# Characteristics and Changes over Time of Alcohol-Related Chronic Liver Diseases in Italy

**DOI:** 10.1155/2018/9151820

**Published:** 2018-09-23

**Authors:** Tommaso Stroffolini, Evangelista Sagnelli, Caterina Sagnelli, Filomena Morisco, Sergio Babudieri, Caterina Furlan, Mario Pirisi, Maurizio Russello, Antonina Smedile, Mariantonietta Pisaturo, Piero Luigi Almasio

**Affiliations:** ^1^Department of Tropical and Infectious Diseases, Policlinico Umberto I, Rome, Italy; ^2^Department of Mental Health and Public Medicine, Campania University Luigi Vanvitelli, Naples, Italy; ^3^Department of Clinical Medicine and Surgery, Gastroenterology Unit, University of Naples “Federico II”, Naples, Italy; ^4^Clinic of Infectious Diseases, University of Sassari, Sassari, Italy; ^5^Department of Translational Medicine, Università del Piemonte Orientale, Novara, Italy; ^6^Liver Unit, Hospital “G. Garibaldi” Catania, Catania, Italy; ^7^Department of Medical Sciences, University of Turin, Turin, Italy; ^8^Unit of Infectious Diseases, S. Anna and S. Sebastian Hospital, Caserta, Italy; ^9^Biomedical Department of Internal Medicine e Specialities (Di.Bi.M.I.S.), University of Palermo, Palermo, Italy

## Abstract

**Introduction:**

To evaluate the characteristics of alcohol-related chronic liver disease (CLD) in Italy and their potential changes over time.

**Patients and Methods:**

Subjects with CLD were enrolled in two national surveys performed in 2001 and in 2014 in Italy. The two surveys prospectively recruited patients aged ≥ 18 years referring to more than 80 Italian liver units scattered all over the country using similar clinical approach, analytical methods, and threshold of risky alcohol intake definition (≥ 3 units/day in men and ≥ 2 units/day in women).

**Results:**

Out of 12,256 enrolled subjects, 2,717 (22.2%) reported a risky alcohol intake. Of them, anti-HCV positive was observed in 48.3% of subjects. The overall sex ratio (M/F) was 3.1, decreasing from 3.8 in 2001 to 1.3 in 2014. Women were significantly older than men (58.9 versus 53.1 years; *p* < 0.01) and an increasing ageing over time was observed in both sexes. The proportion of subjects with liver cirrhosis increased over time in both sexes, and decompensated stage (Child B or C) was detected in 55.9% of cases in 2001 and 46.0% in 2014.

**Conclusions:**

Risky alcohol intake plays a role in more than one-fifth of CLD in Italy, with a shift over time towards an older age and a more severe liver disease stage. These data put alcohol back in the spotlight with an important role in CLD in the years to come in Italy.

## 1. Introduction

Alcohol consumption is a major cause of chronic liver disease, particularly in Western and Eastern Europe, where the highest worldwide per capita intake of alcohol is reported [1, 2]. In the United Kingdom there was a 5-fold increase in cirrhosis mortality among men and a 4-fold increase among women from 1950 through 2000, which parallels the doubled alcohol consumption recorded in these countries over the same period [3]. However, only about 35% of heavy drinkers develop the disease [4], suggesting that, beyond alcohol abuse, other factors are involved in the development of liver cirrhosis [5]. In Italy, a significant decrease in the proportion of risky alcohol consumers has been over time observed, from 21.3% in 2007 to 15.7% in 2015. This downtrend was greater in men (from 30.6% to 23.0%) than in women (from 12.6% to 9.0%) [6].

The wealth on information on hepatitis viruses has decreased the attention paid to the role of alcohol intake in the development of chronic liver disease (CLD) and progression to cirrhosis, so that information on alcoholic liver diseases in Italy is scanty and fragmentary.

Pooling the cases recruited in two Italian national surveys performed in 2001 [7] and in 2014 [8, 9] on the characteristics of subjects with chronic liver diseases (CLD) referring more than 80 liver units, we have evaluated the main aspects of alcohol-related CLD and their potential changes over time.

## 2. Patients and Methods

The two national surveys have been previously described [7–9]. The first one enrolled 9,997 persons with CLD consecutively referring to 79 hospital liver units for a six months' period in 2001. The second one recruited 2,557 CLD cases consecutively referring to 15 hospital liver units in 2014. Subjects enrolled in 2014 were different from those in the 2001 survey. Criteria of enrolment were aged over 18 years and either altered hepatic biochemistry, presence of etiologic markers of liver damage, or history of symptoms consistent with CLD. Both inpatients and outpatients were recruited. In both studies, the enrolling liver units were scattered all over the country. Most of the 15 hospitals participating in the second study had also taken part in the first one. The liver units participating to the two surveys collected the data prospectively had comparable access procedures and used similar approach and similar analytical methods.

Personal data were collected in full compliance with the Italian law on personal data protection, and each patient gave his/her informed consent to participate. All procedures applied in the two studies were in accordance with the international guidelines, with the standards of human experimentation of the local Ethics Committees and with the Helsinki Declaration of 1975, revised in 1983. At the time of the first observation, each patient signed their informed consent for the collection of personal data. Patients who agreed to undergo liver biopsy signed an appropriate informed consent before biopsy was performed. Patients were enrolled only once at their first observation. For each patient, a precoded questionnaire containing demographic, epidemiological, and clinical data was filled out. No patient refused to participate in the studies.

### 2.1. Diagnostic Criteria

In both surveys, the presence of serum hepatitis B surface antigen (HBsAg) identified hepatitis B virus (HBV) aetiology and that of antibody to hepatitis C virus (anti-HCV), an HCV aetiology. Autoimmune chronic hepatitis and primary biliary cholangitis, hereditary hemochromatosis, and Wilson's disease were diagnosed according to standardized international criteria [10–13].

An alcohol intake > 40 g/day for males (≥ 3 drinks a day) and > 30 g/day for females (≥ 2 drinks a day) for at least 5 years was considered an etiologic factor for alcoholic liver disease [14, 15].

Nonalcoholic fatty liver disease (NAFLD/NASH) was diagnosed based on abnormal serum values of alanine aminotransferase (ALT) associated with hepatic steatosis identified by liver histology and/or ultrasound (US), in the absence of other known causes of liver disease [16]. CLD was considered cryptogenic in the absence of any viral, autoimmune, or metabolic aetiology.

Chronic hepatitis was diagnosed based on liver histology, when available, or on the persistence (>6 months) of abnormal ALT in the absence of clinical, biochemical, and US evidence of liver cirrhosis [17]. Liver cirrhosis was diagnosed by liver biopsy (LB) or, in the absence, on the presence of characteristic clinical, biochemical, and ultrasound signs [17]. The diagnosis of hepatocellular carcinoma (HCC) was based on histological and/or imaging findings and alfa-1-fetoprotein serum levels [18].

Percutaneous LB was performed, if requested by the physician in care for diagnostic purposes, under US guidance using a disposable modified Menghini's needle. In each liver unit, a skilled pathologist unaware of the clinical and laboratory data evaluated liver histology. Liver necro-inflammation and fibrosis were assessed by the Ishak [19] or Metavir scoring system [20] and standardized criteria were used to convert the Ishak score to a Metavir scores [21]. Liver biopsy slices were read by local of central pathologist.

### 2.2. Serologic Assays

Serum HBsAg and antibody to HCV were sought using commercial immunoenzymatic assays. Routine tests were applied to seek the etiologic markers of autoimmune hepatitis, primary biliary cholangitis, iron and copper overload, and liver functions.

### 2.3. Statistical Analysis

The data were collected in a preestablished electronic CRF database (web-based data collection, e-CRF provided by Air-Tel®, Airon Telematica, Milan, Italy). Differences in means and in proportion were evaluated by Student's t-test and chi square test or Fisher's exact test, respectively. A *p* value < 0.05 was considered to be significant. All *p* values were two-tailed.

## 3. Results

Out of the 12,754 enrolled subjects with CLD (9,997 in 2001 and 2,557 in 2014), 2,717 (22.2%) reported a risky alcohol intake. The mean age was 54.2 years with a sex ratio (M/F) of 3.2. The mean Body Mass Index (BMI) resulted in 25.6. A nearly similar proportion of cases was observed in northern/central areas (47.7%) and in southern/islands (49.8%). Nearly one-third (35.5%) of subjects had liver cirrhosis without (30.7%) or with (4.5%) HCC. Of these 2,717 patients, 1,313 (48.3%) were also anti-HCV positive, while 1,163 (42.7 %) reported a risky alcohol intake as the only etiologic agent of CLD. The comparison of the two studies shows an over time statistically significant older mean age (53.2 years versus 60.2 years, *p* < 0.001), a decreasing M/F sex ratio (from 3.8 to 1.3, *p* < 0.001), an increasing proportion of cases from northern/central areas (*p* < 0.001), an increase in the proportion of subjects declaring alcohol abuse with a decrease in the proportion of those having alcoholism plus anti-HCV positivity (*p* < 0.02), and, finally, an increase of subjects with liver cirrhosis without or with HCC (39.9% versus 43.4%, *p* < 0.01) (Table 1).

As compared to males, females were over time more likely older (58.0 years versus 53.0 years, *p* < 0.01), were born in northern/central areas (53.5% versus 45.7%, *p* < 0.01), and more frequently declared alcohol abuse (53.5% versus 39.3%, *p* < 0.01). No significant difference by gender was observed according to diagnosis (Table 2).

Statistically significant intragender over time differences were observed in males, with an ageing age, an increasing proportion of cases related to alcohol abuse alone, and an increasing proportion of cirrhotic patients with or without HCC (Table 3). Intragender differences over time were observed even in females with an ageing age, a higher proportion of cases with liver cirrhosis, and a decreasing proportion of subjects with alcohol abuse alone (Table 4).

Characteristics of liver cirrhosis cases are reported in Table 5. The most relevant findings are the over time decrease of both the M/F sex ratio (*p* < 0.001) and the rate of cases (55.9% to 46.8%; *p* < 0.05) with in a decompensated stage (Child B/C).

## 4. Discussion

The present work is a careful subanalysis of data only generically stated in an earlier publication [8], focusing exclusively on alcohol-related CLD. The 2001 and the 2014 nationwide prevalence surveys were structurally similar. Both studies were cross-sectional and prospectively enrolled for a given time inpatients and outpatients aged 18 or more with CLD of any aetiology referring for altered hepatic biochemistry or positivity of hepatitis virus serum markers to one of the participating liver units located all over the country. The same clinical approach, analytical methods, and facilities to access the participating liver units, operating in district general or academic hospitals, have been adopted. In addition, several of these liver units have participated to both 2001 and 2014 surveys. Finally, the same threshold of a risky alcohol intake was adopted in both surveys [14, 15]. Consequently, the pooling and the comparison of the two studies may be no matter of concern. The most striking findings of this large series of subjects with CLD alcohol-related are a male preponderance, an older mean age of women, an increasing ageing over time in both sexes, a high proportion of subjects with a history of alcohol abuse and anti-HCV positive, and the majority of liver cirrhosis cases presenting at a decompensated stage.

The greater vulnerability of women and lower safe limits for alcohol consumption have been long recognized [22]. Women have a higher risk of alcoholic cirrhosis compared to men for a given level of alcohol intake [22, 23]. Some factors may explain this sex difference: lower production of gastric alcohol dehydrogenase and smaller volume of alcohol distribution. In addition, it has been suggested that female hormones increase alcohol mediated liver injury in an unknown fashion [24]. However, males account worldwide for 67% of liver cirrhosis deaths due to alcohol, and worldwide reports (enclosed the present study) have indicated a higher prevalence of alcoholic liver disease in men [2]. This could be because men typically drink more frequently than women do and, more frequently, they are heavy drinkers and alcoholics, regardless of geographic locations [25].

The great decrease of sex ratio from 2001 to 2014 parallels the consistent linear reduction of alcohol intake in the last decade in Italy, particularly among men [6], likely due to both Italian Sanitary Authorities' campaign to prevent alcohol abuse and to economic crisis, which reduces the likelihood of purchase alcohol beverages for money restriction.

The statistically significant younger age of male subjects suggests an earlier in the life exposure to alcohol in this sex. However, the cross-sectional structure of the two studies may have generated a potential survival bias; i.e., men might have died for other diseases more likely than women might do. On the other hand, the biphasic course of liver disease progression in women [26], with a more rapid increase of liver fibrosis in the late menopause than in the reproductive/premenopausal/early menopausal status [27], should be also considered. These explanations are not mutually exclusive, but all may have played a role in the observed different age pattern by sex. In any case, the observed ageing over time in both sexes may suggest an improvement of CLD managing over the past 13 years.

The high proportion of alcoholic and anti-HCV positive subjects reflects both the wide spread of HCV infection occurring in Italy after the world war II and the likelihood of behaviours at higher risk of exposure to this infection in these subjects.

A large case-control study showed that two-thirds of decompensated liver cirrhosis in Italy were attributable to alcohol abuse [28]. This figure, even if overestimated for having the authors inaccurately used the proportion of risky alcohol consumers in the hospital controls instead of in the general population for calculating the Population Attributable Risk [29], reflects the tendency of alcoholics to seek medical care only once symptoms are disclosed.

We acknowledge some potential limitations of the present study. Firstly, the history of alcohol intake was retrospectively recorded so that findings may be influenced by a recall bias and by the tendency of alcoholics to minimize or deny alcohol abuse at the time of observation to avoid the negative stigmata associated with alcoholism.

Secondly, some concern for a potential referral bias exists, since about 50% of patients with alcohol abuse were also anti-HCV positive (this condition that may have favoured the enrolment) and the remaining 50% anti-HCV negative often showed a severe liver disease (i.e., liver cirrhosis), a clinical condition that may have favoured the request for medical assistance and the enrolment in the study. This limitation, however, affects any study focusing on alcoholic CLD, as alcoholics tend to refer to hospitals only once severe symptoms have developed.

Thirdly, the definition of risky alcohol intake used in the present study does not capture the pattern of binge drinking, i.e., the consumption of 5 or more drinks in males or 4 or more drinks in women in about two hours [30], a phenomenon poorly investigated even in other published studies although continuously increasing in western countries [31]. Finally, type of beverage has not been assessed; the abuse in drinking wine has been recently shown to be associated with a lower risk of severe liver disease than the abuse in drinking beer and liquor [32].

In conclusion, this survey evidences that risky alcohol intake plays a role in more than one-fifth of CLD in Italy, with a shift over time towards an older age and a more severe disease stage. Considering the decreasing role of chronic HCV [33] and HBV [34] infections in Italy, these data put alcohol back in the spotlight with an important role in CLD in the years to come.

## Figures and Tables

**Table 1:** Baseline characteristics of 2,717 enrolled subjects with chronic liver disease alcohol related in Italy, by year of recruitment.

**Characteristic**	**Overall**	**Study 2001**	**Study 2014**	**p-value**
	**(n=2,717)**	**(n=2,342)**	**(n=375)**	
**Age, years, mean ± SD**	54.2 ± 12.8	53.2 ± 14.8	60.2 ± 13.4	<0.001

**Sex Ratio (M/F)**	3.1	3.8	1.3	<0.001

**BMI, mean ± SD**	25.6 ± 3.8	25.5 ± 3.8	23.0 ± 4.3	0.015

**Area of birth**				
(i) Northern/Central Italy	1,295 (47.7%)	1,099 (46.9%)	196 (52.3%)	<0.001
(ii) Southern Italy/Islands	1,354 (49.8%)	1,192 (50.9%)	159 (42.4%)	
(iii) Born abroad	71 (2.6%)	51 (2.2%)	20 (5.3%)	

**Risk factors **				
(i) Alcohol intake alone	1,163 (42.8%)	983 (42.0%)	179 (47.7%)	0.02
(ii) Alcohol intake/HBsAg positive	172 (6.3%)	140 (6.0%)	32 (8.5%)	
(iii) Alcohol intake/anti-HCV positivity	1,313 (48.3%)	1,157 (49.4%)	154 (41.1%)	
(iv) Alcohol intake/HBsAg positive/anti-HCV positive	72 (6.3%)	62 (2.6%)	10 (2.7%)	

**Diagnosis**				
(i) Chronic hepatitis	1,762 (64.8%)	1,550 (66.1%)	212 (56.5%)	<0.001
(ii) Liver cirrhosis without HCC	834 (30.7%)	697 (29.8%)	137 (36.5%)	
(iii) Liver cirrhosis with HCC	121 (4.5%)	95 (4.1%)	26 (6.9%)	

BMI: body mass index. HCC: hepatocellular carcinoma.

**Table 2:** Characteristics of 2,717 enrolled subjects with chronic liver disease alcohol related in Italy, by sex.

**Characteristics**	**Males**	**Females**	**Sex Ratio (M/F)**	**p-value**
	**(n=2,059)**	**(n=658)**		
**Age, years,** mean ± SD	53.0 ± 14.7	58.0 ± 14.5	-	<0.001

**BMI, **mean ± SD	25.7 ± 3.7	25.1 ± 4.4	-	0.001

**Area of birth**				
(i) Northern/Central Italy	943 (45.8%)	352 (53.5%)	2.7	<0.001
(ii) Southern Italy/Islands	1,069 (51.9%)	282 (42.9%)	3.8	
(iii) Born abroad	47 (2.3%)	24 (3.6%)	2.0	

**Risk factors **				
(i) Alcohol intake alone	810 (39.3%)	352 (53.5%)	2.3	<0.001
(ii) Alcohol intake/HBsAg positive	147 (7.1%)	25 (3.8%)	5.9	
(iii) Alcohol intake/anti-HCV positivity	1,040 (50.5%)	271 (41.2%)	3.8	
(iv) Alcohol intake/HBsAg positive/anti-HCV positive	62 (3.0%)	10 (1.5%)	6.2	

**Diagnosis**				
(i) Chronic hepatitis	1,348 (65.5%)	414 (62.9%)	3.3	0.15
(ii) Liver cirrhosis without HCC	614 (29.8%)	220 (33.4%)	2.8	
(iii) Liver cirrhosis with HCC	97 (4.7%)	24 (3.6%)	4.0	

BMI: body mass index. HCC: hepatocellular carcinoma.

**Table 3:** Characteristics of chronic liver diseases alcohol related in male sex over time in Italy.

**Characteristics**	**2001 Study **	**2014 Study **	**p-value**
	**(n**°**=1,852)**	**(n**°**=207)**	
**Age (years), **mean ± SD	52.4 ± 14.7	58.0 ± 13.9	<0.001

**BMI **(kg/m^2^), mean ± SD	25.6 ± 3.6	26.4 ± 4.1	<0.05

**Area of origin**			
(i) Northern/Central Italy	888 (45.8%)	95 (45.9%)	0.03
(ii) Southern Italy/Islands	967 (52.2%)	102 (49.3%)	
(iii) Born abroad	37 (2.0%)	10 (4.8%)	

**Risk factors**			
(i) Alcohol intake alone	710 (38.3%)	100 (48.3%)	0.015
(ii) Alcohol intake/HBsAg positive	129 (7.0%)	18 (8.7%)	
(iii) Alcohol intake/anti-HCV positivity	957 (51.7%)	83 (40.1%)	
(iv) Alcohol intake/HBsAg positive/anti-HCV positive	56 (3.0%)	6 (2.9%)	

**Diagnosis**			
(i) Chronic hepatitis	1,231 (66.5%)	117 (56.5%)	0.004
(ii) Liver cirrhosis without HCC	541 (29.2%)	73 (35.3%)	
(iii) Liver cirrhosis with HCC	80 (4.3%)	17 (8.2%)	

BMI: body mass index. HCC: hepatocellular carcinoma.

**Table 4:** Characteristics of chronic liver diseases alcohol related in female sex over time, in Italy.

**Characteristics**	**2001 Study **	**2014 Study **	**p-value**
	**(n**°**=490)**	**(n**°**=168)**	
**Age (years),** mean ± SD	56.3 ± 14.8	63.0 ± 12.3	<0.001

**BMI **(kg/m^2^), mean ± SD	24.9 ± 4.3	25.6 ± 4.4	0.1

**Area of origin**			
(i) Northern/Central Italy	251 (51.2%)	101 (60.1%)	0.009
(ii) Southern Italy/Islands	225 (45.9%)	57 (33.9%)	
(iii) Born abroad	14 (2.9%)	10 (6.0%)	

**Risk factors**			
(i) Alcohol intake alone	273 (55.7%)	79 (47.0%)	0.002
(ii) Alcohol intake/HBsAg positive	11 (2.2%)	14 (8.3%)	
(iii) Alcohol intake/anti-HCV positivity	200 (40.8%)	71 (42.3%)	
(iv) Alcohol intake/HBsAg positive/anti-HCV positive	6 (1.2%)	4 (2.4%)	

**Diagnosis**			
(i) Chronic hepatitis	319 (65.1%)	95 (56.5%)	0.09
(ii) Liver cirrhosis without HCC	156 (31.8%)	64 (38.1%)	
(iii) Liver cirrhosis with HCC	15 (3.1%)	9 (5.4%)	

BMI: body mass index. HCC: hepatocellular carcinoma.

**Table 5:** Characteristics of 955 subjects with alcohol-related liver cirrhosis in Italy, by year of recruitment.

**Characteristics**	**Overall**	**Study 2001**	**Study 2014**	**p-value**
	**(n**°**=955)**	**(n**°**=792)**	**(n**°**=163)**	
**Age (years), mean ± SD**	60.0 ± 12.3	59.8 ± 12.1	60.7 ± 13.4	0.4

**Sex Ratio (M/F)**	2.9	3.6	1.3	<0.001

**BMI, mean ± SD**	25.8 ± 4.1	25.7 ± 4.0	25.7 ± 4.0	0.3

**Area of origin**				
(i) Northern/Central Italy	423 (44.2%)	349 (44.1%)	74 (45.4%)	0.005
(ii) Southern Italy/Islands	510 (53.3%)	429 (54.2%)	79 (48.5%)	
(iii) Born abroad	24 (2.5%)	14 (1.8%)	10 (6.1%)	

**Risk factors**				
(i) Alcohol intake alone	459 (48.0%)	374 (47.2%)	85 (52.1%)	0.7
(ii) Alcohol intake/HBsAg positive	63 (6.6%)	54 (6.8%)	9 (5.5%)	
(iii) Alcohol intake/anti-HCV positivity	407 (42.5%)	340 (42.9%)	65 (39.9%)	
(iv) Alcohol intake/HBsAg positive/anti-HCV positive	28 (2.9%)	24 (3.0%)	4 (2.5%)	

**Diagnosis**				
(i) Liver cirrhosis without HCC	836 (87.4%)	697 (88.0%)	137 (84.0%)	0.2
(ii) Liver cirrhosis with HCC	121 (12.6%)	95 (12.0%)	26 (16.0%)	

**Child-Pugh class**				
(i) A	381 (45.6%)	307 (44.1%)	74 (54.0%)	0.04
(ii) B/C	455 (54.4%)	390 (55.9%)	63 (46.0%)	

BMI: body mass index. HCC: hepatocellular carcinoma.

## Data Availability

The demographic, epidemiological and clinical data used to support the findings of this study are included within the article.

## Abbreviations

HCV: Hepatitis C virus
HCC: Hepatocellular carcinoma
anti-HCV: Antibody to HCV
HCV RNA: Hepatitis C virus ribonucleic acid
HBV: Hepatitis B virus
HbsAg: Hepatitis B surface antigen
CLD: Chronic liver disease
PCR: Polymerase chain reaction
ALT: Alanine aminotransferase
US: Ultrasound.
